# Statins and postmenopausal breast cancer risk; results from the KARMA cohort

**DOI:** 10.1007/s10552-026-02156-x

**Published:** 2026-04-03

**Authors:** Marie Klintman, Ann H. Rosendahl, Benjamin R. Johannesen, Deirdre Cronin-Fenton, Mikael Eriksson, Kamila Czene, Per Hall, Signe Borgquist

**Affiliations:** 1 Department of Clinical Sciences Lund, Oncology, Lund University, Skåne University Hospital, 221 85 Lund, SE Sweden; 2 Department of Clinical Epidemiology, Aarhus University, Aarhus, Denmark; 3 Department of Medical Epidemiology and Biostatistics, Karolinska Institute, Stockholm, Sweden; 4 Department of Oncology, Södersjukhuset, Stockholm, Sweden; 5 Department of Oncology, Aarhus University Hospital, Aarhus University, Aarhus, Denmark

**Keywords:** Statins, Overweight, Obesity, BMI, Breast cancer risk, Breast cancer subtypes

## Abstract

**Purpose:**

To study the incidence and subtype of breast cancer in relation to incident and prevalent statin use in a contemporary Swedish prospective cohort, The Karolinska Mammography Project for Risk Prediction of Breast Cancer, KARMA.

**Methods:**

A total of 35,315 postmenopausal women attending mammography and included in the KARMA cohort (Jan 2011–March 2013) with data on statin use and potential confounders were studied. During eight years of follow-up, 785 incident invasive breast cancer cases were identified.

**Results:**

A total of 16% of women were prevalent statin users (prior to study inclusion) and 9% were incident statin users (following study inclusion). In multivariable Cox regression analyses, there was no significant association between incident or prevalent statin use and risk of incident breast cancer (HR_adj_ 1.24, 95% CI 0.89–1.72, and HR_adj_ 0.90, 95% CI 0.73–1.11, respectively). Similarly, no significant association was found for incident or prevalent statin use and subtype-specific risk of breast cancer.

**Conclusion:**

This prospective population-based study performed in a modern screening population with a substantial number of statin users, concurs with previous publications showing no evidence of an association between statin use and risk of postmenopausal breast cancer.

## Introduction/background

Statins, or 3-Hydroxy-3-methylglutaryl coenzyme A (HMG-CoA) inhibitors are widely used lipid-lowering drugs in the primary and secondary prevention of cardiovascular disease. They may also play a role in breast cancer [1]. Apart from a cardioprotective effect of statins, early statin studies suggested a link between statin use and increased risk of breast cancer [2]. This was supported by studies in rodents suggesting a tumor promoting effect of statins, although with treatment doses far exceeding maximum doses in humans [3]. However, the bulk of preclinical data on statins shows suppressive, pleiotropic effects on tumor growth in breast cancer cell lines and animal models [4, 5].

Statins inhibit the mevalonate pathway, reducing cholesterol synthesis and the production of isoprenoids that regulate small GTPase signaling [6]. Through these mechanisms, statins may influence cancer-related processes such as cell proliferation, invasion, and metastasis potential [7], apoptosis [8], angiogenesis [9], and inflammation [7, 10]. Nevertheless, their anti-tumor efficacy in vivo may be limited by bioavailability, with hydrophilic statins largely confined to hepatic tissue, whereas lipophilic statins may possibly demonstrate greater potential due to wider tissue distribution [5].

Research suggests that statin use may be associated with reduced risk of contralateral breast cancer [11], recurrence and mortality in breast cancer [12–17]. Preclinical data indicate statins may affect breast cancer progression differently depending on subtype. In ER-positive disease statins may modulate ER activity and downstream signaling [18], whereas in HER2-enriched cancers, disruption of HER2-associated membrane domains may inhibit HER-signaling and enhance the efficacy of HER2-targeted therapies[18]. Still, the potential primary preventive effect of statins in clinical studies on breast cancer risk is less clear. Some early studies suggested increased breast cancer incidence [2, 19–22], and some found a reduced risk or no association [23–26] according to statin use. Meta-analyses based on studies published from 1996 to 2016 do not support an association between statin use and incidence of breast cancer [27–30]. However, as overweight and obesity—which is associated with a higher risk of developing breast cancer and higher cancer mortality [31, 32]—have increased over the years, a larger proportion of the population is now prescribed statins. Studies were therefore needed to outline the association of statins with breast cancer risk today in the era of mammography screening. Lastly, there are few clinical studies on statin use and possible associations with subtype-specific breast cancer [7, 33]. To further address these questions in a modern cohort we therefore aimed to study the effect of statin use and the risk of incident breast cancer as well as subtype of breast cancer, in postmenopausal women in the KARMA (KArolinska Mammography Project for Risk Prediction of Breast Cancer) cohort [34]—a well-defined prospective Swedish contemporary cohort of over 70,000 women enrolling participants in recent time.

## Methods

### Study population

**Fig. 1:**
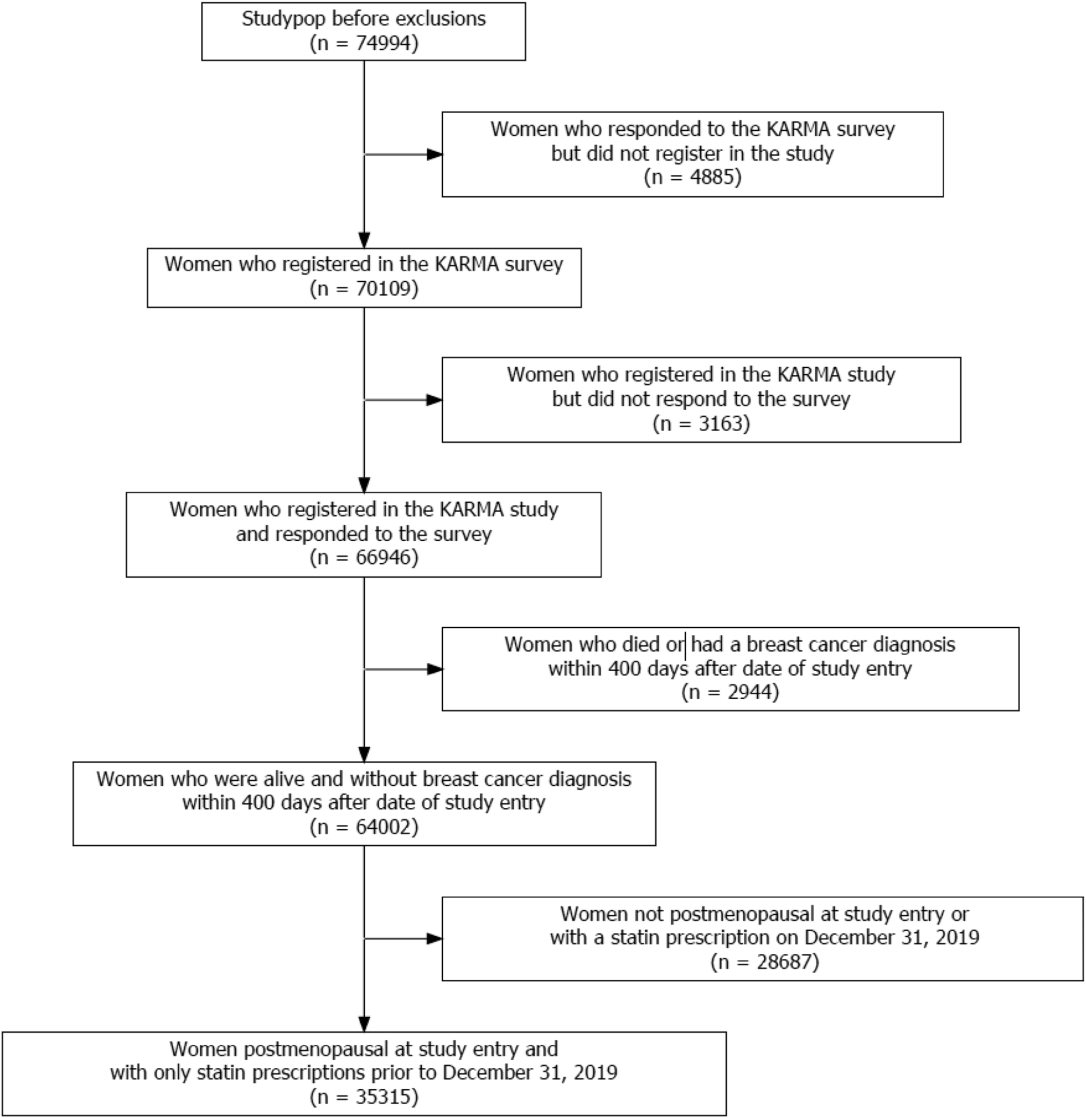
Study flow diagram

Between January 2011 and March 2013, all women undergoing clinical or screening mammography at one of four hospitals in Sweden (Södersjukhuset, Stockholm, Helsingborg Hospital, Skåne University Hospital, and Landskrona Hospital) were invited to participate in the KARMA study (http://karmastudy.org) [34]. The study, including a total of 74,994 Swedish women, focused on individualized prevention and screening with the aim of reducing the incidence and mortality in breast cancer. At study inclusion, the participants signed informed consent and answered detailed web-based lifestyle questionnaires, and donated blood samples. The study also included permission to access data from Swedish national health registers on pathology and treatment data. The KARMA population has been described in detail previously [35], and a study flow diagram of the study population is presented in Fig. 1. Women who were diagnosed with breast cancer or died within 400 days of study entry were excluded to ensure that statin exposure could be consistently defined during the exposure assessment period. This approach avoids misclassification of exposure status and ensures that exposure determination precedes the start of follow-up for all participants. The study cohort was restricted to postmenopausal women, among whom statin use is more frequently prescribed compared to premenopausal women. Data on menopausal status were derived from the questionnaire. In short, 35,315 postmenopausal women were included in the present study, and during follow-up, 785 patients were diagnosed with incident breast cancer; of these, 729 patients had full information on variables used in the adjusted models. The study was approved by the ethical committee of the Karolinska Institute (# 2017/958).

### Data collection and classification

The Swedish National Prescribed Drug Register was used to access data on statin use, insulin, and metformin co-medication [36], and use of hormonal contraception and hormone replacement therapy was retrieved from the KARMA questionnaire. The national Swedish Cancer Register [37] was used to identify all cancer diagnoses, the Cause of Death Register [38] to identify causes of death, and lastly linkage to breast cancer-specific NKBC (National Quality Register of Breast Cancer) registers [39] to retrieve patient and pathological data for the incident cases including age at diagnosis, tumor size (≤ 20 mm, vs > 20 mm), nodal status (positive/negative), Nottingham Histological Grade (III versus I + II), estrogen receptor (ER)-, progesterone receptor (PR)-, and human epidermal growth factor receptor-2 (HER2)-status (positive/negative), and Ki67 (% positive cells, with < 10%, 11–20%, and > 20% defined as low, intermediate, or high, respectively). The definition of breast cancer subtypes was based on the Swedish national guidelines. Immunohistochemical-based surrogate markers were used with Luminal A defined as ER + HER2- with either (i) histological grade I (irrespective of Ki67), or (ii) histological grade II with low Ki67, or (ii) histological grade II, intermediate Ki67 and PR > 20%. Luminal B was defined as ER + HER2 + and either (i) histological grade III (irrespective of Ki67) (ii) histological grade II and high Ki67, or (iii) histological grade II, intermediate Ki67, and PR < 20%. Basal-like was defined as ER, PR, and HER2-negative, and HER2-enriched as HER2 + irrespective of ER-status.

### Statin use

Information on statin prescriptions was ascertained from the Swedish National Prescribed Drug Register, which dates back to 2005 [36]. Prevalent use of statins was defined as at least one statin prescription with an Anatomic Therapeutic Chemical code (ATC code) beginning with “C10AA” before study entry. Prevalent users were defined as women who had filled two statin prescriptions within 400 days of each other and had at least one statin prescription prior to study entry, representing ongoing or prior use. These participants were excluded from the incident-user analysis to maintain mutually exclusive exposure groups.

Incident users were defined as women who initiated statin therapy after study entry, with no prior statin prescriptions recorded. To confirm treatment initiation and avoid immortal time bias, incident statin use was treated as a time-varying exposure: women were considered statin users 400 days after their first post-entry prescription, provided that a second prescription was filled within 400 days of the first. Participants with only one post-entry prescription remained classified as unexposed unless a second prescription was redeemed within this 400-day window.

The 400-day maximum interval between prescriptions was chosen to capture all likely statin users, as statins may be dispensed for up to one year at a time. Statin exposure was lagged by 400 days to reduce bias from increased medical surveillance among statin users and to allow sufficient biological exposure time. Women diagnosed with breast cancer or who died within 400 days of study entry were excluded to ensure consistent exposure classification before follow-up began and to avoid misclassification.

In sensitivity analyses, statin exposure was restricted to lipophilic statins (atorvastatin, simvastatin, lovastatin, fluvastatin, and cerivastatin).

### Co-variates

Lifestyle and reproductive health factors associated with both risk of breast cancer and statin use included Body Mass Index (BMI), use of hormonal contraception, hormone replacement therapy, smoking and alcohol, were retrieved from the KARMA-questionnaires. Data on co-medications included insulin (ATC code A10A) and metformin (ATC code A10B) and were derived from the Swedish National Prescribed Drug Register. Co-medication was defined as one prescription between study entry and 365 days before study entry.

### Statistical Methods

We considered statin exposure as prevalent and incident statin use, analyzed in two separate models. The first model evaluated prevalent users, while the second model evaluated incident use, focusing on incident statin use as a time-varying exposure. In the model for prevalent statin use, participants were followed from the date of inclusion in the KARMA study, while in the model for incident statin use participants were followed from 400 days after inclusion in the KARMA study. In both models, participants were followed until the date of invasive breast cancer diagnosis, date of death, or December 31, 2019, whichever came first.

In the main analysis, a lag period of 400 days from the first prescription to the start of follow-up was used. Three separate sensitivity analyses were performed (i) a longer lag period of 800 days from the first prescription to the start of follow-up, (ii) 400 days from the second prescription to the start of follow-up, and last (ii) including the use of lipophilic statins only. Cumulative incidence of invasive breast cancer with regard to (i) all incident breast cancer and (ii) breast cancer defined by known prognostic variables (defined by TNM, [tumor size, node status, metastases], age at diagnosis, histological grade, and expression of ER, PR, and HER2), and (iii) subtype-specific breast cancer defined by the immunohistochemical surrogate markers ER, PR, HER2, and Ki67 with death as a competing risk was calculated using the Aalen–Johansen estimator. The cumulative incidence was estimated using the Aalen–Johansen estimator and including competing events as competing events instead of just as censoring events. Hazard ratios (HRs) with 95% confidence intervals (CI) for (i) all incident invasive breast cancer, (ii) breast cancer defined by known prognostic variables, and (iii) subtype-specific breast cancer was calculated using a Cox proportional hazards model with time on study as the underlying time scale. Cause-specific hazard ratios were estimated with ordinary Cox regression including competing endpoints as censoring events. The multivariable model was adjusted for age (spline), BMI (spline), use of oral contraceptives (yes/no), hormone replacement therapy (HRT; yes/no), use of co-medications insulin and metformin (yes/no), and lifestyle factors that included smoking (pack years categorical, three levels, never/previous/current), and alcohol (yes/no, and grams per week). Age at inclusion, BMI and alcohol were incorporated into the model as natural cubic splines with four knots. Given the small number of exposed cases in some subgroups, we examined alternative spline specifications to assess model stability; however, the overall conclusions remained unchanged. The proportionality assumption was checked visually by inspection of the log minus log of the survival curve based on the Kaplan–Meier estimator, and no violation was found. All statistical analyses were carried out in R version 4.1.2.

## Results

### Patient and tumor characteristics

Baseline characteristics on all participants (*n* = 35,315) are presented in Table 1. Only participants who had full information on all factors used in the adjusted models were included in the subsequent analyses (*n* = 33,049). Median age at study entry was 62 years (Inter Quartile Range; IQR 57–67). A total of 25% of the study population were statin users (any use, *n* = 9,428 simvastatin, and *n* = 44,851 atorvastatin), where 9% were incident users and 16% were prevalent users.

**Table 1:** Baseline characteristics in relation to statin use in the 35,315 postmenopausal patients in the KARMA cohort

Variable	Overall	Non-user	Prevalent	Incident
No of women (%)	35 315 (100)	26 487 (75%)	5644 (16%)	3184 (9%)
Age at entry, years (median [IQR])	62 [57, 67]	61 [56, 66]	66 [61, 69]	64 [59, 68]
Age at entry, years (%)				
≤ 29	0 (0.0)	0 (0.0)	0 (0.0)	0 (0.0)
30–39	16 (0.0)	16 (0.1)	0 (0.0)	0 (0.0)
40–49	1134 (3.2)	1065 (4.0)	29 (0.5)	40 (1.3)
50–59	12 385 (35.1)	10 542 (39.8)	988 (17.5)	855 (26.9)
60–69	17 344 (49.1)	12 184 (46.0)	3384 (60.0)	1776 (55.8)
70–79	4412 (12.5)	2666 (10.1)	1234 (21.9)	512 (16.1)
≥ 80	24 (0.1)	14 (0.1)	9 (0.2)	1 (0.0)
Height, cm (median [IQR])	166 [162, 170]	166 [162, 170]	165 [161, 169]	165 [161, 169]
Weight, kg (median [IQR])	68 [62, 77]	68 [61, 75]	71 [64, 80]	70 [63, 79]
BMI at entry, kg/m2 (median [IQR])	24.8 [22.6, 27.7]	24.5 [22.3, 27.1]	26.2 [23.7, 29.4]	25.7 [23.3, 28.7]
Age at menarche, years (median [IQR])	13 [12, 14]	13 [12, 14]	13 [12, 14]	13 [12, 14]
Age at menarche, missing (%)	1258 (3.6)	929 (3.5)	214 (3.8)	115 (3.6)
Age at menopause, years (median [IQR])	50 [47, 53]	50 [48, 53]	50 [47, 53]	51 [47, 54]
Age at menopause, missing (%)	18 110 (51.3)	13 698 (51.7)	2882 (51.1)	1530 (48.1)
No. of pregnancies (%)				
0	3048 (8.8)	2293 (8.8)	502 (9.1)	253 (8.1)
1	3918 (11.4)	2928 (11.3)	640 (11.6)	350 (11.3)
2	11 337 (32.8)	8366 (32.3)	1867 (34.0)	1104 (35.5)
3	8637 (25.0)	6474 (25.0)	1404 (25.6)	759 (24.4)
≥ 4	7576 (21.9)	5854 (22.6)	1082 (19.7)	640 (20.6)
Births (%)				
0	4320 (12.5)	3297 (12.7)	655 (11.9)	368 (11.9)
1	5211 (15.1)	3880 (15.0)	858 (15.6)	473 (15.2)
2	16 002 (46.4)	11 972 (46.2)	2546 (46.3)	1484 (47.8)
3	7071 (20.5)	5320 (20.5)	1135 (20.7)	616 (19.8)
≥ 4	1908 (5.5)	1445 (5.6)	300 (5.5)	163 (5.3)
Age at first childbirth (%)				
≤ 20	4185 (12.1)	2830 (10.9)	890 (16.2)	465 (15.0)
> 20- ≤ 25	11 284 (32.7)	8158 (31.5)	2006 (36.5)	1120 (36.1)
> 25- ≤ 30	9616 (27.9)	7437 (28.7)	1394 (25.4)	785 (25.3)
> 30	5094 (14.8)	4185 (16.2)	546 (9.9)	363 (11.7)
Nulliparous	4320 (12.5)	3297 (12.7)	655 (11.9)	368 (11.9)
Age at first childbirth, years (median [IQR])	25 [22, 29]	26 [23, 29]	24 [21, 27]	25 [21, 28]
No. of women using oral contraceptives (%)				
No	6679 (19.7)	4742 (18.6)	1307 (24.3)	630 (20.8)
Yes	27 235 (80.3)	20 769 (81.4)	4064 (75.7)	2402 (79.2)
No. of women using HRT (%)				
No	19 706 (57.2)	15 221 (58.8)	2825 (51.6)	1660 (53.7)
Yes	14 756 (42.8)	10 679 (41.2)	2648 (48.4)	1429 (46.3)
Breast cancer in family (%)				
No	28 715 (84.6)	21 599 (84.6)	4560 (84.5)	2556 (84.5)
Yes	5233 (15.4)	3926 (15.4)	838 (15.5)	469 (15.5)
No. of women smoking (%)				
Never	14 142 (41.1)	10 763 (41.6)	2207 (40.3)	1172 (37.8)
Previous	15 977 (46.4)	11 917 (46.1)	2612 (47.6)	1448 (46.8)
Current	4326 (12.6)	3186 (12.3)	663 (12.1)	477 (15.4)
Smoking, packyears (median [IQR])	2.00 [0.00, 10.5]	1.80 [0.00, 9.50]	3.00 [0.00, 13.00]	3.00 [0.00, 13.00]
No. of women drinking alcohol (%)				
No	6597 (19.3)	4682 (18.2)	1271 (23.4)	644 (20.9)
Yes	27 613 (80.7)	21 012 (81.8)	4171 (76.6)	2430 (79.1)
Alcohol, gram per week (median [IQR])	37.00 [6.00, 67.00]	37.00 [9.00, 67.00]	37.00 [6.00, 64.00]	37.00 [6.00, 68.00]
No. of women using insulin (%)				
No	34 892 (98.8)	26 422 (99.8)	5334 (94.5)	3136 (98.5)
Yes	423 (1.2)	65 (0.2)	310 (5.5)	48 (1.5)
No. of women using metformin (%)				
No	34 469 (97.6)	26 362 (99.5)	5025 (89.0)	3082 (96.8)
Yes	846 (2.4)	125 (0.5)	619 (11.0)	102 (3.2)

In Table 2, patient and tumor characteristics of the 785 incident breast cancer patients are displayed. Hereof, 127 patients were considered prevalent and 44 were incident statin users, respectively. The median age at diagnosis was 68.0 years (IQR 63.0–71.0), median BMI 25.1 kg/m^2^ (IQR 22.8–27.7), and median tumor size 14.0 mm (IQR 10.0–20.0). Regarding tumor characteristics, 23.4% of the patients had lymph node positive disease, 79.0% of tumors were ER + , 61.0% PR + , 9.6% HER2 + , 22.2% were histological grade III, and 39.2% were highly proliferating according to Ki67. Based on immunohistochemical surrogate markers for subtyping, 71.2% were luminal-like (ER + /HER2-), 44.4% were Luminal-A-like, and 22.9% Luminal-B-like. 7.0% were ER + /HER2 + , 2.2% ER-/HER2 + , and 7.3% were diagnosed with TNBC.

**Table 2:** Patient- and tumor characteristics of the 785 women diagnosed with an incident breast cancer

Variable	All	Non-user	Prevalent	Incident
Overall	785	614	127	44
Age at diagnosis (median [IQR])	68 [63, 71]	68 [62, 71]	69 [65, 72]	69 [64, 71]
Tumor size, mm (median [IQR])	14 [10, 20]	14 [10, 20]	13.5 [10, 20]	14 [11, 20.5]
Tumor size (%) T0	1 (0.1)	1 (0.2)	0 (0.0)	0 (0.0)
T1 (1–20 mm)	519 (66.1)	409 (66.6)	84 (66.1)	26 (59.1)
T2 (21–50 mm)	154 (19.6)	127 (20.7)	19 (15.0)	8 (18.2)
T3 (> 50 mm)	24 (3.1)	15 (2.4)	7 (5.5)	2 (4.5)
T4	1 (0.1)	0 (0.0)	1 (0.8)	0 (0.0)
Missing	86 (11.0)	62 (10.1)	16 (12.6)	8 (18.2)
Nodal status (%) Negative	517 (65.9)	403 (65.6)	89 (70.1)	25 (56.8)
Positive	184 (23.4)	149 (24.3)	22 (17.3)	13 (29.5)
Missing	84 (10.7)	62 (10.1)	16 (12.6)	6 (13.6)
ER status (%) Negative	75 (9.6)	60 (9.8)	13 (10.2)	2 (4.5)
Positive	620 (79.0)	486 (79.2)	98 (77.2)	36 (81.8)
Missing	90 (11.5)	68 (11.1)	16 (12.6)	6 (13.6)
PR status (%) Negative	209 (26.6)	172 (28.0)	31 (24.4)	6 (13.6)
Positive	479 (61.0)	369 (60.1)	79 (62.2)	31 (70.5)
Missing	97 (12.4)	73 (11.9)	17 (13.4)	7 (15.9)
HER2 status (%) Negative	621 (79.1)	485 (79.0)	101 (79.5)	35 (79.5)
Positive	75 (9.6)	64 (10.4)	8 (6.3)	3 (6.8)
Missing	89 (11.3)	65 (10.6)	18 (14.2)	6 (13.6)
Histological grade (%) 1	155 (19.7)	121 (19.7)	24 (18.9)	10 (22.7)
2	337 (42.9)	264 (43.0)	60 (47.2)	13 (29.5)
3	174 (22.2)	141 (23.0)	23 (18.1)	10 (22.7)
Missing	119 (15.2)	88 (14.3)	20 (15.7)	11 (25.0)
Ki67 (%) High	308 (39.2)	253 (41.2)	39 (30.7)	16 (36.4)
Intermediate	103 (13.1)	77 (12.5)	23 (18.1)	3 (6.8)
Low	281 (35.8)	215 (35.0)	48 (37.8)	18 (40.9)
Missing	93 (11.8)	69 (11.2)	17 (13.4)	7 (15.9)
ER + /HER2- (%) No	129 (16.4)	107 (17.4)	17 (13.4)	5 (11.4)
Yes	559 (71.2)	434 (70.7)	92 (72.4)	33 (75.0)
Missing	97 (12.4)	73 (11.9)	18 (14.2)	6 (13.6)
ER + /HER2 + (%) No	633 (80.6)	494 (80.5)	104 (81.9)	35 (79.5)
Yes	55 (7.0)	47 (7.7)	5 (3.9)	3 (6.8)
Missing	97 (12.4)	73 (11.9)	18 (14.2)	6 (13.6)
ER-/HER2 + (%) No	671 (85.5)	527 (85.8)	106 (83.5)	38 (86.4)
Yes	17 (2.2)	14 (2.3)	3 (2.4)	0 (0.0)
Missing	97 (12.4)	73 (11.9)	18 (14.2)	6 (13.6)
TNBC (%) No	631 (80.4)	495 (80.6)	100 (78.7)	36 (81.8)
Yes	57 (7.3)	46 (7.5)	9 (7.1)	2 (4.5)
Missing	97 (12.4)	73 (11.9)	18 (14.2)	6 (13.6)
Luminal A (%) No	293 (37.3)	239 (38.9)	38 (29.9)	16 (36.4)
Yes	347 (44.2)	266 (43.3)	65 (51.2)	16 (36.4)
Missing	145 (18.5)	109 (17.8)	24 (18.9)	12 (27.3)
Luminal B (%) No	464 (59.1)	365 (59.4)	79 (62.2)	20 (45.5)
Yes	180 (22.9)	144 (23.5)	24 (18.9)	12 (27.3)
Missing	141 (18.0)	105 (17.1)	24 (18.9)	12 (27.3)

### Prevalent and incident statin use and risk of breast cancer

The median follow-up time was 2,412 days (6.6 years). Table 3 displays the risk of incident breast cancer in relation to prevalent and incident statin use. Among the prevalent statin users (n = 5,213) there were 118 incident breast cancer cases. The 8-year cumulative risk of incident breast cancer (95% CI) was 2.38% (2.17%–2.59%) and 2.34% (1.94%–2.79%) for non-users and prevalent users, respectively. There was no evidence of an association with prevalent statin use and risk of incident breast cancer in either the crude (HR 1.04, 95%CI 0.85–1.27) or adjusted model (HR_adj_ 0.90, 95% CI 0.73–1.11).

**Table 3:** Crude rates per 1,000 person years, 8-year cumulative risk, crude and adjusted* hazard ratios for breast cancer in relation to prevalent and incident statin use

	Persons	Cases	Person years	Crude rate per 1,000 person years (95% CI)	8-year cumulative risk (95% CI)	Crude HR (95% CI)	Adjusted HR (95% CI)
Prevalent statin use							
No	27 836	611	180 000	3.39 (3.13–3.67)	2.38% (2.17%-2.59%)	1 (reference)	1 (reference)
Yes	5213	118	33 400	3.53 (2.92–4.23)	2.34% (1.94%-2.79%)	1.04 (0.85–1.27)	0.90 (0.73–1.11)
Incident statin use							
No	27 839	571	171 000	3.34 (3.07–3.63)	2.35% (2.14%-2.58%)	1 (reference)	1 (reference)
Yes	2947	40	9290	4.31 (3.08–5.86)	3.33% (2.26%-4.73%)	1.43 (1.04–1.99)	1.24 (0.89–1.72)

^*^Adjusted for age (spline), bmi (spline), oral contraceptives (binary), HRT use (binary), smoking status (never/previous/current), alcohol (indicator variable), alcohol (spline), insulin (binary), and metformin (binary)

Among the incident statin users (n = 2,947), a total of 40 incident breast cancers were detected, and an increased risk of breast cancer was observed among incident statin users compared with non-users in the crude model (HR 1.42, 95%CI 1.04–1.99), but when adjusting the model for relevant co-variates including age, BMI, use of HRT or oral contraceptives, anti-diabetic medications (insulin and metformin), and selected lifestyle factors (smoking and alcohol), the effect attenuated (HR_adj_ 1.24, 95% CI 0.89–1.72), however with the point estimate and confidence interval overlapping that of the crude model. For the following analyses, only incident use was considered and multivariate results presented.

### Sensitivity analyses

In the three separate sensitivity analyses, no associations were found between incident statin use and breast cancer risk. In the first model, the lag period was extended to 800 days from the day of the first prescription (HR_adj_ 0.98, 95% CI 0.63–1.52), in the second model, the lag period of 400 days was defined from the second prescription date (HR_adj_ 1.25, 95% CI 0.90–1.73), and, lastly, an analysis considering lipophilic statin use (HR_adj_ 1.22, 95% CI 0.88–1.70) (Table 4).

**Table 4:** Sensitivity analyses and crude rates per 1,000 person years, 8-year cumulative risk, crude and adjusted* hazard ratios for breast cancer in relation to prevalent or incident statin use with (i) an extended lag period of 800 days (ii) delaying start av lag period to second prescription (iii) lipophilic statins only

	Persons	Cases	Person years	Crude rate per 1,000 person years (95% CI)	8-year cumulative risk (95% CI)	Crude HR (95% CI)	Adjusted HR (95% CI)
Incident statin use extended lag period of 800 days							
No	27 627	457	143 000	3.19 (2.9–3.49)	1.92% (1.72%-2.13%)	1 (reference)	1 (reference)
Yes	2338	21	6390	3.28 (2.03–5.02)	1.77% (1.07%-2.78%)	1.14 (0.74–1.78)	0.98 (0.63–1.52)

	Persons	Cases	Person years	Crude rate per 1,000 person years (95% CI)	8-year cumulative risk (95% CI)	Crude HR (95% CI)	Adjusted HR (95% CI)
Incident statin use delayed start of lag period to 400 days defined from the second prescription date							
No	27 927	576	172 000	3.36 (3.09–3.64)	2.36% (2.15%-2.59%)	1 (reference)	1 (reference)
Yes	2877	40	9130	4.38 (3.13–5.97)	3.41% (2.33%-4.81%)	1.46 (1.05–2.02)	1.25 (0.90–1.73)

	Persons	Cases	Person years	Crude rate per 1,000 person years (95% CI)	8-year cumulative risk (95% CI)	Crude HR (95% CI)	Adjusted HR (95% CI)
Incident statin use lipophilic statins only							
No	27 839	571	171 000	3.34 (3.07–3.63)	2.35% (2.14%-2.58%)	1 (reference)	1 (reference)
Yes	2891	39	9140	4.26 (3.03–5.83)	3.31% (2.23%-4.71%)	1.42 (1.02–1.97)	1.22 (0.88–1.70)

### Incident statin use and risk of subtype-specific breast cancer

In Table 5, the risk of incident breast cancer based on the tumor-related prognostic factors such as tumor size, nodal status, ER, PR, HER2, histological grade or Ki67 in relation to statin use is displayed. Due to the few breast cancers in each subgroup, results for many of the models are not included. For the remaining models, no associations were found between incident statin use and risk of breast cancer based on tumor size, node status, PR expression, histological grade or HER2 and Ki67 expressions.

**Table 5:** Crude rates per 1,000 person years, 8-year cumulative risk, and crude and adjusted* hazard ratios for known prognostic breast cancer variables in relation to incident statin use

	Postmenopausal patients							
Variable	Incident statin use	Persons	Cases	Person years	Crude rate per 1,000 person years (95% CI)	8-year cumulative risk (95% CI)	Crude HR (95% CI)	Adjusted HR (95% CI)
Tumor size < 20 mm	No	27 839	380	171 000	2.22 (2.01–2.46)	1.59% (1.41%-1.79%)	1 (reference)	1 (reference)
	Yes	2947	22	9290	2.37 (1.48–3.59)	2.08% (1.22%-3.32%)	1.16 (0.75–1.78)	1.03 (0.66–1.59)
Tumor size > 20 mm	No	27 839	132	171 000	0.77 (0.65–0.92)	0.56% (0.45%-0.68%)	NA	NA
	Yes	2947	10	9290	1.08 (0.52–1.98)	0.62% (0.31%-1.15%)	NA	NA
Nodal status Negative	No	27 839	373	17 1000	2.18 (1.97–2.42)	1.57% (1.39%-1.76%)	NA	NA
	Yes	2947	22	9290	2.37 (1.48–3.59)	1.80% (1.06%-2.88%)	NA	NA
Nodal status Positive	No	27 839	140	171 000	0.82 (0.69–0.97)	0.58% (0.48%-0.70%)	1 (reference)	1 (reference)
	Yes	2947	12	9290	1.29 (0.67–2.26)	1.02% (0.50%-1.89%)	1.82 (1.00–3.31)	1.54 (0.84–2.83)
ER status Positive	No	27 839	451	171 000	2.64 (2.4–2.89)	1.88% (1.69%-2.10%)	NA	NA
	Yes	2947	33	9290	3.55 (2.45–4.99)	2.59% (1.69%-3.78%)	NA	NA
ER status Negative	No	27 839	57	171 000	0.33 (0.25–0.43)	0.25% (0.19%-0.33%)	NA	NA
	Yes	2947	1	9290	0.11 (0.0027–0.6)	0.21% (0.02%-1.13%)	NA	NA
PR status Positive	No	27 839	347	171 000	2.03 (1.82–2.26)	1.48% (1.30%-1.68%)	1 (reference)	1 (reference)
	Yes	2947	29	9290	3.12 (2.09–4.48)	2.52% (1.60%-3.79%)	1.68 (1.14–2.47)	1.43 (0.97–2.11)
PR status Negative	No	27 839	157	171 000	0.92 (0.781–1.07)	0.64% (0.54%-0.75%)	NA	NA
	Yes	2947	4	9290	0.431 (0.117–1.1)	0.20% (0.07%-0.49%)	NA	NA
HER2 status Negative	No	26 491	474	193 000	2.45 (2.23–2.68)	1.89% (1.72%-2.07%)	1 (reference)	1 (reference)
	Yes	5019	90	36 400	2.47 (1.99–3.04)	1.86% (1.51%-2.28%)	1.01 (0.81–1.27)	0.88 (0.69–1.12)
HER2 status Positive	No	26 491	64	193 000	0.33 (0.26–0.42)	0.25% (0.19%-0.32%)	1 (reference)	1 (reference)
	Yes	5019	9	36 400	0.25 (0.11–0.47)	0.18% (0.09%-0.34%)	0.75 (0.37–1.50)	0.70 (0.33–1.46)
Histological grade 1/2	No	27 839	358	171 000	2.09 (1.88–2.32)	1.52% (1.34%-1.72%)	1 (reference)	1 (reference)
	Yes	2947	21	9290	2.26 (1.4–3.46)	1.72% (1.01%-2.75%)	1.18 (0.76–1.84)	1 (0.64–1.57)
Histological grade 3	No	27 839	129	171 000	0.76 (0.63–0.90)	0.53% (0.44%-0.63%)	NA	NA
	Yes	2947	9	9290	0.97 (0.44–1.84)	0.62% (0.26%-1.32%)	NA	NA
Ki67 Low	No	27 839	275	171 000	1.61 (1.42–1.81)	1.16% (1.01%-1.33%)	1 (reference)	1 (reference)
	Yes	2947	18	9290	1.94 (1.15–3.06)	1.65% (0.91%-2.78%)	1.3 (0.8–2.1)	1.1 (0.67–1.78)
Ki67 High	No	27 839	234	171 000	1.37 (1.2–1.56)	0.98% (0.84%-1.13%)	1 (reference)	1 (reference)
	Yes	2947	15	9290	1.62 (0.90–2.66)	1.09% (0.59%-1.88%)	1.29 (0.76–2.19)	1.16 (0.68–1.98)

^*^adjusted for age (spline), bmi (spline), oral contraceptives (binary), HRT use (binary), smoking status (never/previous/current), alcohol (indicator variable), alcohol (spline), insulin (binary), and metformin (binary)

Last, in Table 6 the risk of subtype-specific breast cancer based on immunohistochemical surrogate markers and statin use is displayed. Similarly, due to low numbers of cases in subgroups, only the overall luminal (ER + /HER2-) and luminal A groups could be considered, as the other models did not converge. In univariable analysis, an increased risk of ER + /HER2- breast cancer was noticed, but it was not retained in the multivariable-adjusted model (crude and adjusted HRs were 1.51 (95% CI 1.04–2.19) and 1.30 (95% CI 0.89–1.90), respectively). No association was found for risk of luminal A cancer and statin use (HR_adj_ 0.92, 95% CI 0.53–1.58).

**Table 6:** Crude rates per 1,000 person years, 8-year cumulative risk, crude and adjusted* hazard ratios for immunohistochemical surrogate marker subtype-specific breast cancer in relation to incident statin use

	Postmenopausal patients							
Subtype	Incident statin use	Persons	Cases	Person years	Crude rate per 1,000 person years (95% CI)	8-year cumulative risk (95% CI)	Crude HR (95% CI)	Adjusted HR (95% CI)
ER + , HER2-	No	27 839	403	171 000	2.36 (2.13–2.6)	1.70% (1.51%-1.90%)	1 (reference)	1 (reference)
	Yes	2947	30	9290	3.23 (2.18–4.61)	2.44% (1.56%-3.62%)	1.51 (1.04–2.19)	1.30 (0.89–1.90)
ER + , HER2 +	No	27 839	43	171 000	0.25 (0.18–0.34)	0.17% (0.12%-0.23%)	NA	NA
	Yes	2947	3	9290	0.32 (0.067–0.94)	0.15% (0.04%-0.42%)	NA	NA
ER-, HER2 +	No	27 839	13	171 000	0.076 (0.041–0.13)	0.05% (0.03%-0.08%)	NA	NA
	Yes	2947	0	9290	0 (0–0.40)	0.00% (NA-NA)	NA	NA
TNBC	No	27 839	44	171 000	0.26 (0.19–0.35)	0.20% (0.14%-0.28%)	NA	NA
	Yes	2947	1	9290	0.11 (0.0027–0.60)	0.21% (0.02%-1.13%)	NA	NA
Luminal A	No	27 839	251	171 000	1.47 (1.29–1.66)	1.06% (0.91%-1.23%)	1 (reference)	1 (reference)
	Yes	2947	14	9290	1.51 (0.82–2.53)	1.30% (0.67%-2.32%)	1.12 (0.65–1.93)	0.92(0.53–1.58)
Luminal B	No	27 839	132	171 000	0.77 (0.65–0.92)	0.56% (0.46%-0.69%)	NA	NA
	Yes	2947	12	9290	1.29 (0.67–2.26)	0.68% (0.37%-1.17%)	NA	NA

^*^adjusted for age (spline), bmi (spline), oral contraceptives (binary), HRT use (binary), smoking status (never/previous/current), alcohol (indicator variable), alcohol (spline), insulin (binary), and metformin (binary)

## Discussion

In this large, contemporary prospective Swedish cohort of postmenopausal women, we found no association between statin use—neither prevalent nor incident use—and breast cancer risk when adjusting for relevant confounders. This validates findings from meta-analyses of previous studies including randomized, observational, and case–control studies [27–29], suggesting statins to be safe medications not affecting breast cancer initiation.

The in vitro evidence for the effect of statins and cancer development is conflicting, as disruption of the mevalonate pathway exerted by statins may have both pro- and anticancer effects [3, 9]. Statins may reduce tumor cell progression and carcinogenesis by lowering LDL levels required for rapidly dividing cell membrane synthesis [40], and by reducing prenylation and subsequent GTPase cell signaling required for tumor growth [41]. However, due to differences in bioavailability, solubility, and potential pleiotropic effects of hydrophilic and lipophilic statins, we also performed a sensitivity analysis addressing lipophilic statin use separately. The results were similar, with a suggested increased risk of breast cancer in the crude model, which attenuated in the multivariable-adjusted model. However, it also had a point estimate and confidence interval overlapping that of the crude model.

Also, less is known of the effects of long-term dysregulation of the mevalonate pathway and lowering of cholesterol levels in a clinical setting. Even though results on the effect of statin use and risk of breast cancer over the years have been conflicting, with some studies indicating an increased risk [2, 19–21], others a reduced risk [23, 24], three meta-analyses have concluded no associations [27]. In studies with a positive association between statin use and breast cancer risk, results suggest the duration of statin use may have affected the risk. In the McDougal paper, only women using statins for ≥ 10 years had a 72% higher risk of invasive ductal carcinoma and an 82% higher risk of invasive lobular carcinoma, whereas there was no association with breast cancer risk in ever-users [19]. In contrast to this, another study identified that short-term users (less than 180 days) had an increased risk in contrast to participants with statin use of > 2 years, who displayed a significantly lower risk of breast cancer compared to non-users, although numbers in the study were small. For patients who used both statins and HRT, only long-term HRT-users had an increased risk of breast cancer [20]. Last, in a smaller observational study, the association between short-term (< 4 years) lipophilic statin use and breast cancer risk was limited to women with obesity and PR-negative disease only [21]. The 2017 meta-analysis instead suggested a duration effect with a reduced risk with longer duration of statin use [27], which has further support in a study with reduced risks of contralateral breast cancer, specifically in long-term statin users [11]. The number of patients receiving statins has steadily increased over the years, with the first studies on statin use and risk of breast cancer having a frequency of less than 10% users, whereas in our study 25% of the participants were statin users. The mean duration of statin use is only considered possible to clearly define for the incident users, and the present study is limited by the follow-up of 6.4 years, which does not allow for assessments of short versus long-term use. With more users and longer follow-up, the observed effects of statin use on breast cancer risk may still change.

As for associations between statin use and risk of subtype-specific breast cancer, a study by Kumar and colleagues demonstrated a lower frequency of ER- and PR- tumors among statin users compared with non-users [42]. Similarly, in a study assessing statin use and risk of contralateral breast cancer, statin users with a primary breast cancer being ER- had a significantly reduced risk of developing contralateral breast cancer compared to non-statin users, implying a secondary preventive or adjuvant effect [11]. The present study could not validate these findings or associations between statin use and risk of subtype-specific tumors. However, as the estimates were imprecise due to small numbers, these findings may be considered as exploratory only.

Some tumor characteristics among the prevalent statin users differed from those among non-users and incident users. However, the numbers in the present study were low and the estimates were imprecise and should be interpreted with caution. There were more node-negative and luminal A tumors among the prevalent user cohort compared with the non-users and incident users, features generally associated with a better prognosis and lower risk of recurrence. Both prevalent and incident statin use were examined to account for potential differences between long-term users and new initiators. As prevalent users may represent a selected subgroup of survivors and adherent patients, prevalent user bias could partly explain the observed differences between the two analyses.

These data may be mirrored in evidence from studies showing that statin users have a reduced risk of contralateral breast cancer [11], a better prognosis [12, 13], and also a reduced risk of distant, but not loco-regional, recurrences [43].

The strengths of this study are that incident statin data rely on national prescription registers and not on recall, which may otherwise bias results. The true statin use would therefore be expected to be reliable. However, the National Prescribed Drug Register in Sweden started in 2005, and prescriptions and possible prevalent use pre-dating 2005 are not available. Therefore, an unknown number of patients who used statins before 2005 may have been recorded as non-users. We also have data on and have been able to adjust for relevant confounders related to risk of both breast cancer and statin use, which might otherwise bias results.

The limitations of the study are the small number of breast cancers among incident statin users, leading to a lack of precision of our estimates. Further, although we have records of prescriptions, the actual consumption of the drugs is unknown. There may also be an unknown bias in systematical difference in adherence between patient groups*.* However, as individuals had to pay a proportion of the cost of their prescriptions, they were likely to consume the medication. Both prevalent and incident statin use were examined to account for potential differences between long-term users and new initiators. As prevalent users may represent a selected subgroup of survivors and adherent patients, prevalent user bias could partly explain the observed differences between the two analyses. We furthermore defined statin use as at least two prescriptions to minimize this potential bias. Also, there is no consensus in studies on the choice of the length of the lag period and time of exposure in relation to cancer risk. By choosing a lag period of 400 days from the first prescription, we ensured at least one year of exposure to the drug before studying the effects on breast cancer risk. The results were also further strengthened in the sensitivity analyses by first extending the lag period to 800 days, allowing for a longer time of exposure, and second by starting the 400-day lag period from the date of the second prescription. These analyses did not change the main results with one notable exception where the HR_adj_ was 0.98 for the extended lag period compared with 1.24 and 1.25 for the main analysis and the delayed start, respectively. This may suggest underlying differences in time exposed and breast cancer risk. Last, the follow-up of 6.4 years is potentially too short to assess the possible carcinogenic effects of statin exposure as the latency between exposure to a potential carcinogen and cancer development may be up to 20 years [44]. However, results from the prevalent users point in the same direction with no association with breast cancer risk.

In summary, as overweight and related health care issues such as hyperlipidemia and use of cholesterol-lowering medication are steadily increasing, this study adds to the literature supporting the safe use of statins in relation to risk of breast cancer. In this modern cohort of postmenopausal women with 25% statin users, there was no increased risk of developing breast cancer when adjusting for relevant confounders. Future studies with longer follow-up to assess possible long-term effects on risk are, however, still warranted to further address the use of statins in relation to risk of cancer.

## Data Availability

The datasets generated during and/or analyzed during the current study are not publicly available due to GDPR regulations, but full de-identifiable data are available from the corresponding author on reasonable request.
